# Marital Strain and Support and Subjective Well-Being in Later Life: Ascribing a Role to Early-Life Conditions

**DOI:** 10.1093/geroni/igab046.1571

**Published:** 2021-12-17

**Authors:** Laura Upenieks, Yingling Liu

**Affiliations:** 1 University of Texas at San Antonio, San Antonio, Texas, United States; 2 Baylor University, Waco, Texas, United States

## Abstract

Decades of research have the beneficial effects of marital support and the detrimental consequences of marital strain on health and well-being. However, we know relatively less about how circumstances in childhood—a key developmental period of the life course—influence the relational structure in which later life is embedded and any implications this may hold for well-being. We integrate the life course perspective with the stress process model to offer a framework for how childhood conditions (childhood happiness, family structure, and financial strain) moderate the relationship between marital support/strain and subjective well-being in older adulthood in potentially different ways for men and women. The consequences of marital strain may be more severe and the benefits of marital support may not be as strongly felt for those adults who experienced greater adversity during childhood. Drawing on longitudinal data from Waves 2 (2010-2011) and 3 (2015-2016) of the NSHAP project (N = 1,376), results from lagged dependent variable models suggest that marital support buffers the effect of not living with both parents in childhood on subjective well-being for men. Meanwhile, women raised in families that experienced financial hardship reported lower subjective well-being in the context of marital strain in later life. No significant interaction effects were obtained for childhood happiness. Taken together, our findings suggest that adverse experiences in childhood can be scarring, particularly in the context of strained intimate relationships. However, a supportive marriage can, in some cases, offset the effects of childhood hardship on subjective well-being in later life.

